# Supplementary material for: Content-rich biological network constructed by mining PubMed abstracts
Source: BMC Bioinformatics. 2004 Oct 8;5:147. doi: 10.1186/1471-2105-5-147 (PMC528731; doi:10.1186/1471-2105-5-147)
Supplement: Additional File 5 — The original Chilibot query results of the term "long-term potentiation (LTP)" and 22 other terms, limiting the latest references analyzed to the years 1990, 1995, 2000, and 2004. [file 1471-2105-5-147-S5.bz2 › chilibotAdditionalFile5/ltp2000/html/LTP_NMDA.html]

 


 **LTP** and **NMDA** 
  
Found 972 abstracts in PubMed,  **30 abstracts were retrieved and analyzed**.  


---

 Search Google  |
 PDF files only 
|  EDU domain only 

---

**Interactive relationship** (e.g. stimulation, inhibition, etc)

- Thus  **LTP** ... observed upon blockade of adenosine A 1 receptors is dependent upon  **NMDA**  receptor activation.  Ref: 10978579 Neurosci Lett, 2000
- Here we show that, in the CA1 region of the hippocampus, reduction of postsynaptic calcium influx by partial blockade of  **NMDA**  N methyl D aspartate receptors results in a conversion of  **LTP**  to LTD and a loss of input specificity normally associated with  **LTP** , with LTD appearing at heterosynaptic inputs.  Ref: 11117745 Nature, 2000
- Theta burst induced  **LTP**  was larger in the presence of the selective adenosine A 1 receptor antagonist, dipropyl 8 cyclopentylxanthine DPCPX, 50nM, 40.5 6.6% increase in fEPSP than in the control solution 18.2 4.7% increase, and was completely prevented in the presence of DPCPX 50nM plus the selective  **NMDA**  receptor antagonist, DL 2 amino 5 phosphonopentanoate AP 0microM, 3.3 7.0% change.  Ref: 10978579 Neurosci Lett, 2000
- These results further indicate that mGlu receptor mediated potentiation of  **NMDA**  responses is not essential for the induction of  **LTP** .  Ref: 10991916 Br J Pharmacol, 2000
- Recent studies indicate that the molecular mechanism of behavioral experience induced structural plasticity in the adult brain differs from that of the developing brain, and can be disassociated from the  **NMDA**  mediated long term potentiation  [ **LTP** ]   **LTP**  phenomenon.  Ref: 11075831 Hippocampus, 2000
- This observation suggests that defective  **NMDA**  receptor mechanisms at the Schaffer collateral CA 1 pyramidal cell synapse cannot explain age related  **LTP**  induction deficits that are observed under some stimulation protocols.  Ref: 11016529 Neurobiol AgingNeurobiol Aging, 2000
- The induction of graded burst discharges in magnesium free conditions and the expression of burst discharges evoked in normal bathing medium with subsequent disinhibition were both blocked by DL 2 amino 4 phosphonovaleric acid APV and were therefore  **NMDA**  receptor dependent, in contrast to long term potentiation  [ **LTP** ]   **LTP**  in the perforant path, which is induced by  **NMDA**  receptors and is also expressed by alpha amino 3 hydroxy 5 methyl 4 isoxazoleproprionate AMPA receptors.  Ref: 11110816 J Neurophysiol, 2000
- The STA mediated stimulation of  **NMDA**  receptors was found to promote adaptive reactions moderate activation of IRS, long term potentiation  [ **LTP** ]  of synaptic transmission in the postanoxic period.  Ref: 11055198 Vestn Ross Akad Med Nauk, 2000
- This  **LTP**  did not require the activation of postsynaptic gamma aminobutyric acid A GABA A receptors but depended on the activation of  **NMDA**  receptors.  Ref: 10980014 J Neurophysiol, 2000
- This persistent suppression of  **LTP**  was not secondary to the long lasting changes in  **NMDA**  receptor activation, because the isolated  **NMDA**  receptor mediated responses did not show a long term enhancement in response to a 30 min magnesium free ACSF application.  Ref: 10980002 J Neurophysiol, 2000
- **NMDA**  receptor dependent plasticity of granule cell spike generation, which can be distinguished from  **LTP**  and induces long term susceptibility to epileptic burst discharge under conditions of reduced inhibition, could modify information processing in the hippocampus and promote epileptic synchronization by increasing excitatory input into CA3.  Ref: 11110816 J Neurophysiol, 2000
- In conclusion, IFN alpha induced inhibition of  **LTP**  is, at least in part, mediated by a postsynaptic effect, by tyrosine kinase activity, and by non  **NMDA**  glutamate receptors.  Ref: 11121525 Brain Res, 2000
- The N methyl D aspartate  **NMDA**  subtype of glutamate receptors in the mammalian brain plays a central role in synaptic plasticity underlying refinement of neuronal connections during development, or processes like long term potentiation  [ **LTP** ]   **LTP** , learning and memory.  Ref: 11003045 Life Sci, 2000

**Parallel relationship** (e.g. studied together, co-existance, homology, etc.)

- The role of the  **NMDA**  receptor NMDAR in long term potentiation  [ **LTP** ]   **LTP**  is now well established.  Ref: 11020211 J Neurosci Res, 2000
- The possibility that protein kinase C PKC is involved in the induction of N methyl D aspartate  **NMDA**  receptor dependent long term potentiation  [ **LTP** ]   **LTP**  at CA1 synapses in the hippocampus has been the subject of considerable investigation.  Ref: 11069602 Eur J Neurosci, 2000
- Here, we investigated the role of D1 and D2 receptors in this  **NMDA**  receptor dependent  **LTP** .  Ref: 11069975 J Neurosci, 2000
- To further assess the complex interplay between DA and  **NMDA**  receptors, changes in extracellular DA levels in the PFC were estimated during  **LTP** , and a significant increase was observed immediately after tetanus.  Ref: 11069975 J Neurosci, 2000
- Long term potentiation  [ **LTP** ]  in the presence of  **NMDA**  receptor antagonist arylalkylamine spider toxins.  Ref: 11020211 J Neurosci Res, 2000
- Protein synthesis inhibitors block the maintenance of  **NMDA**  receptor dependent long term potentiation  [ **LTP** ]   **LTP**  both in vivo and in vitro.  Ref: 11069960 J Neurosci, 2000
- Essential role of D1 but not D2 receptors in the  **NMDA**  receptor dependent long term potentiation  [ **LTP** ]  at hippocampal prefrontal cortex synapses in vivo.  Ref: 11069975 J Neurosci, 2000
- Under conditions where the released glutamate can activate  **NMDA**  receptors, long term potentiation  [ **LTP** ]   **LTP**  of the excitatory inputs is induced.  Ref: 10985354 Neuron, 2000
- Long term potentiation  [ **LTP** ]  of the late  **NMDA**  dependent components of neuron responses in the cat motor cortex to stimulation of the direct cortical input from field 5 of the parietal cortex.  Ref: 10981945 Neurosci Behav PhysiolNeurosci Behav Physiol, 2000
- These results suggest that the increase of calcium influx through  **NMDA**  channels and L type VOCCs in turn triggering a PKC dependent signaling cascade is a possible cellular basis underlying this seizure like activity induced inhibition of  **LTP** .  Ref: 10980002 J Neurophysiol, 2000
- These changes in receptor number might underlie selected age associated changes in sensitivity to drugs that influence hippocampal function as well as to changes in  **NMDA**  dependent long term potentiation  [ **LTP** ] .  Ref: 11121523 Brain Res, 2000
- Synapses in the dorsomedial anterior striatum exhibited a propensity to express an  **NMDA**  receptor dependent form of  **LTP**  across the entire developmental time period examined.  Ref: 10980015 J Neurophysiol, 2000
- Therefore, the positive negative post tetanic calcium shift in relation to a previous calcium rise must cause  **NMDA**  dependent  **LTP**  LTDi LTD LTPi or  **NMDA**  independent LTD LTPi  **LTP**  LTDi.  Ref: 11084240 BiosystemsBiosystems, 2000
- However, HFS in dorsolateral striatum from P12 P14 elicited an N methyl D aspartate  **NMDA**  receptor dependent form of  **LTP** .  Ref: 10980015 J Neurophysiol, 2000
- It is based on the assumption that, in diverse central nervous system structures, long term potentiation  [ **LTP** ]  depression  **LTP**  LTD of excitatory transmission depression potentiation of inhibitory transmission, LTDi LTPi is the result of an increasing decreasing the number of phosphorylated AMPA and  **NMDA**  GABA A receptors.  Ref: 11084240 BiosystemsBiosystems, 2000
- In fact, at the perforant path granule cell synapse of rats  **NMDA**  receptor mediated responses are reduced, suggesting a possible change in the threshold for  **LTP**  induction at this synapse.  Ref: 11016529 Neurobiol AgingNeurobiol Aging, 2000
- A novel, competitive mGlu 5 receptor antagonist LY344545 blocks DHPG induced potentiation of  **NMDA**  responses but not the induction of  **LTP**  in rat hippocampal slices.  Ref: 10991916 Br J Pharmacol, 2000
- In human subjects who show a morphologically intact hippocampus that is not the primary seizure focus,  **NMDA**  receptor dependent long term potentiation  [ **LTP** ]   **LTP**  and forskolin induced long lasting potentiation are readily induced at the perforant path dentate gyrus synapse.  Ref: 10995855 J Neurosci, 2000
